# Documenting fall episodes: a scoping review

**DOI:** 10.3389/fpubh.2023.1067243

**Published:** 2023-05-02

**Authors:** Mafalda Pernes, Inês Agostinho, Rafael A. Bernardes, Júlio Belo Fernandes, Cristina Lavareda Baixinho

**Affiliations:** ^1^Nursing School of Lisbon, Lisbon, Portugal; ^2^The Health Sciences Research Unit, Nursing (UICISA:E), Nursing School of Coimbra (ESEnfC), Coimbra, Portugal; ^3^Escola Superior de Saúde Egas Moniz, Caparica, Almada, Portugal; ^4^Grupo de Patologia Médica, Nutrição e Exercício Clínico (PaMNEC)—Centro de Investigação Interdisciplinar Egas Moniz (CiiEM), Almada, Portugal; ^5^Nursing Research, Innovation and Development Cetre of Lisbon (CIDNUR), Lisbon, Portugal

**Keywords:** accidental falls, aged, documentation, clinical audit, recurrent falls, risk

## Abstract

Documentation is an important measure for the management of fall risk because it concentrates the attention of professionals, raises awareness of the existence of fall risk factors, and promotes action to eliminate or minimize them. This study aimed to map the evidence on information to document episodes of falls in older adults. We opted for a scoping review, which followed the Joanna Briggs Institute protocol for this kind of study. The research question that guided the research strategy was “What recommendations emerge from the research on the documentation of falls of the older person?” The inclusion criteria defined were older adults who had at least one fall; nursing documentation after a fall has occurred; and nursing homes, hospitals, community, and long-term care. The search was performed on the following platforms: MEDLINE, CINAHL, Scopus, and Cochrane Database of Systematic Reviews in January 2022 and allowed the identification of 854 articles, which after analysis resulted in a final sample of six articles. The documentation of fall episodes should answer the following questions: Who? What? When? Where? How? Doing what? What was said? What were the consequences? and What has been done? Despite the recommendations for the documentation of fall episodes as a preventive measure for their recurrence, there are no studies evaluating the cost-effectiveness of this measure. Future studies should explore the association between fall documentation, fall recurrence prevention programs, and their impact on the prevalence rate of the second and subsequent falls, as well as the severity of injuries and fear of falling.

## 1. Introduction

Falls are a public health problem for older adults, not only because of their high prevalence in this age group (1–19) but also because of the negative impact they have on functionality, increasing the number of emergency room visits, hospitalizations, co-morbidity, decreased average life expectancy, and mortality rate above 65 years (1–6). Being a transversal accident in the entire context of the life of older adults is more than three times more common in institutionalized older adults (10–16, 18). Other studies have shown that 50.2% of residents suffered at least one fall in the previous year (10, 14), with an average of 1.3 ± 0.48 falls (12) per 1.57 ± 2.78 residents (9).

The cumulative effect of fear of falls, post-fall syndrome, and secondary injuries in a society with an exponential increase in people with neurological disorders and older adults may become epidemic and lead to a consumption of health resources (14). This takes authors to consider falls as a geriatric syndrome not only for people who suffer fractures and who do not regain the functionality they had before falling but also because ptophobia, or fear of falling, leads patients or their caregivers to make some restrictions on activity that conditionate the independence in self-care, over protecting their relative, and making them progressively more dependent on the execution of daily living activities (7–9, 14).

It is unanimous that a cost-effective measure for the prevention of falls is risk identification but also the association of preventive measures appropriate to the individual risk of each person (6–8, 15, 16). A literature review that aimed at identifying the tools used to assess fall risk in institutionalized older adults concludes that the tools with higher predictive value include scales, functional assessment tests, and the question “Did you fall in the last 12 months?” (8). It should be enhanced that this issue has a strong predictive value for the occurrence of new episodes (8), given that the recurrent fall has a variable incidence between 13 and 66.3% (7, 17, 18).

Thus, identifying people who have fallen is crucial because falling is a significant risk for the recurrence of this accident and determining its pattern. In addition, this identification will allow for targeted preventive measures to modifiable risk factors and adopt safety behaviors (14, 19). International recommendations observe that the determination of older adults at greater risk of falling is the first step toward the prevention and, even in those who have already fallen, the determination of the fall mechanism is vital for the implementation of measures for the prevention of recurrent falls (1, 4, 6, 10, 19).

There is a scarce investigation on documentation of episodes of falls and their recurrence. Although there is a recommendation to record all fall episodes, including near-falls, in clinical practice, it is common to identify many episodes of falls that are not witnessed or are witnessed by healthcare professionals and are not recorded in patients' clinical records (8, 9, 14, 19). This issue is even more evident in the community, where most fall episodes occur at the person's home and are not communicated to health professionals. Damián et al. (16) also raise the possibility of underreporting of fall episodes in their study and consider that this underreporting is lower in cases of falls with severe injuries (16). Regarding documentation, when assessing a program to prevent falls in a hospital environment, a team of researchers affirmed that the use of documentation systems, such as care plans and self-records for reporting accidents, can be prevention measures, because they concentrate the attention of professionals, raise awareness for the existence of risk factors of fall, and promote action to eliminate or minimize them (15). Furthermore, prevention protocols recommend that after a fall, healthcare professionals should make a complete evaluation of each incident, with documentation and reporting of all associated factors, following the guidelines of the institution on what measures should be put into practice immediately after a fall (14, 16, 19). Therefore, considering this evidence, this study aimed to map the evidence on information to document episodes of falls in older adults.

## 2. Materials and methods

### 2.1. Study design

Considering the state of the art on the subject and the aim of the study, we opted for conducting a scoping review (SR). This method rapidly maps the key concepts underpinning a research area and the main sources and types of evidence available (20, 21). We followed the Joanna Briggs Institute (JBI) protocol for this type of study (20), which included six steps: (1) identification of the review question, (2) designation of the inclusion and exclusion criteria of studies and identification of relevant studies, (3) selection of the studies, (4) assessment of the level of evidence of the collected literature, according to Joanna Briggs Institute (JBI) guidelines, (5) discussion of the results, and (6) synthesis and presentation of the results obtained (20–22). According to the acronym PCC, the research question was “What recommendations emerge from the research on the documentation of falls in older adults?”

### 2.2. Eligibility criteria

The object of study and the research question guide the definition of the eligibility criteria for the studies included in this SR.

The inclusion criteria defined were as follows:

P—(Population) older adult who had at least one fall;

C—(Concept) nursing documentation after a fall has occurred;

C—(Context) nursing homes, hospitals, community, and long-term care.

The acceptance of studies focusing on the documentation of fall episodes, alone or in association with other interventions, was predefined. Exclusion criteria were studies on the documentation of falls in children and adults and studies on the documentation of risk factors and/or preventive measures of the first fall.

A time limit of 2016 to 2021 was set. This choice is because an initial exploratory search revealed a vast research publication on the topic under study, and researchers are looking for the most current information.

### 2.3. Data collection

The search was performed on the EBSCOhost platforms (MEDLINE and CINAHL databases), Scopus, and Cochrane Database of Systematic Reviews, in January 2022, for studies in Portuguese, English, and Spanish. First, a search was conducted using Health Sciences descriptors DeCS/MeSH, using keywords built from natural language relative to the theme. Table 1 shows the strategy used in Medline.

**Table 1:** Search strategy.

	**Search Strategy**	**Number of manuscripts**
#1	“Elderl*”“[Title/Abstract] OR ““aged”“[Title/Abstract] OR Age**”“[Title/Abstract] OR Older Person *”“[Title/Abstract] OR Older Adult *”“[Title/Abstract] OR ((““Aged”“[Mesh]) OR ““Frail Elderly”“[Mesh])) OR Frail Older Adults ““[Mesh] OR Frail Older Adult ““[Mesh] NOT ““animals”“[Mesh]))	389,305
#2	“(((((((((((((((falls[Title/Abstract]) OR (Accidental falls[Title/Abstract])) OR (fal*[Title/Abstract])) OR (recurrent falls[Title/Abstract])) OR (recurring fal*[Title/Abstract])) OR (new fall[Title/Abstract])) OR (secondary fall[Title/Abstract])) OR (frature after fall[Title/Abstract])) OR (fall injury[Title/Abstract]))) ) OR (accident[MeSH Terms])) OR (accidental fall[MeSH Terms])) OR (accidental falls[MeSH Terms])) OR (falls, accidental[MeSH Terms])”„,""“falls”“[Title/Abstract] OR ”“accidental falls”“[Title/Abstract] OR ”“fal”“[Title/Abstract] OR ”“recurrent falls”“[Title/Abstract] OR ”“recurring fal*”“[Title/Abstract] OR ”“new fall”“[Title/Abstract] OR ”“secondary fall”“[Title/Abstract] OR (”“frature”“[All Fields] AND ”“after fall”“[Title/Abstract]) OR ”“fall injury”“[Title/Abstract] OR ”“accidents”“[MeSH Terms] OR ”“accidental falls”“[MeSH Terms] OR ”“accidental falls”“[MeSH Terms] OR ”“accidental falls”“[MeSH Terms]”	241,018
#3	“((((((((((((((((documentation[Title/Abstract]) OR (doc*[Title/Abstract])) OR (register[Title/Abstract])) OR (reg*[Title/Abstract])) OR (report[Title/Abstract])) OR (rep*[Title/Abstract])) OR (clinical process[Title/Abstract])) OR (nursing process[Title/Abstract])) OR (nursing diagnosis[Title/Abstract])) OR (nurs* doc*[Title/Abstract])) OR (nurs*reg*[Title/Abstract])) OR (hospital registrar[MeSH Terms])) OR (education, post registration nursing[MeSH Terms])) OR (documentation[MeSH Terms])) OR (documentations[MeSH Terms])) OR (audit, clinical[MeSH Terms])) OR (clinical practice guideline[MeSH Terms])”„,""“documentation”“[Title/Abstract] OR ”“doc”“[Title/Abstract] OR ”“register”“[Title/Abstract] OR ”“reg”“[Title/Abstract] OR ”“report”“[Title/Abstract] OR ”“rep”“[Title/Abstract] OR ”“clinical process”“[Title/Abstract] OR ”“nursing process”“[Title/Abstract] OR ”“nursing diagnosis”“[Title/Abstract] OR (”“nurs*”“[All Fields] AND ”“doc”“[Title/Abstract]) OR ”“medical staff, hospital”“[MeSH Terms] OR ”“education, nursing, continuing”“[MeSH Terms] OR ”“documentation”“[MeSH Terms] OR ”“documentation”“[MeSH Terms] OR ”“clinical audit”“[MeSH Terms] OR ((”“nephron clin pract”“[Journal] OR ”“clin pract lond”“[Journal] OR (”“clinical”“[All Fields] AND ”“practice”“[All Fields]) OR ”“clinical practice”“[All Fields]) AND ”“guidelines as topic”“[MeSH Terms])”	3,113,047
#4	#1 AND #2 AND #3	251

In databases, the descriptors were operationalized using the expressions OR and AND. The search codes were constructed using these expressions.

Afterward, the research was extended to Google Scholar, repositories of theses, and official sites of associations/organizations of reference in the area to identify guidelines and gray literature on the subject.

Data extraction was carried out by two reviewers independently (MP and IA), and doubts and disagreements were resolved with the inclusion of a third reviewer (CLB).

### 2.4. Data processing and analysis

During the extraction phase, the content of the articles was thoroughly analyzed, allowing not only to answer the research question but also to understand whether the studies explored the fall risk and its prevalence, as well as the recurrence of interventions focusing on the documentation of falls.

In order to record the contents extracted from the articles in the final bibliographic sample, researchers elaborated a table using MS Excel that included the following items: identification of the title of the article/document; author(s), publication year, type of article; methods and main results/conclusions. The results of the articles that allowed answering the research question were extracted and submitted to narrative synthesis.

Two reviewers carried out data analysis independently, and all research team members validated the narrative synthesis.

### 2.5. Ethical issues

This study includes one of the work packages of a research study on the management of fall risk in older adults that was authorized by an Ethics Committee (PARECER No. CE/IPLEIRIA/46/2020). This is a secondary study that followed the principles of integrity in research. The problem formulation adhered to the principles of clarity, precision, objectivity, and delimitation, allowing its results to contribute to the resolution of a prevalent problem in healthcare, benefiting not only people with neurological diseases but also other older adults at risk of falling.

The study protocol was followed rigorously to ensure the validity of the study. The extraction and analysis of data from the primary study constituents of the bibliographic sample were done with evident respect for the research and results obtained by the other researchers. The reference of the authors who supported the elaboration of this article also followed the recommendations of good academic and scientific practice.

## 3. Results

**Figure 1:**
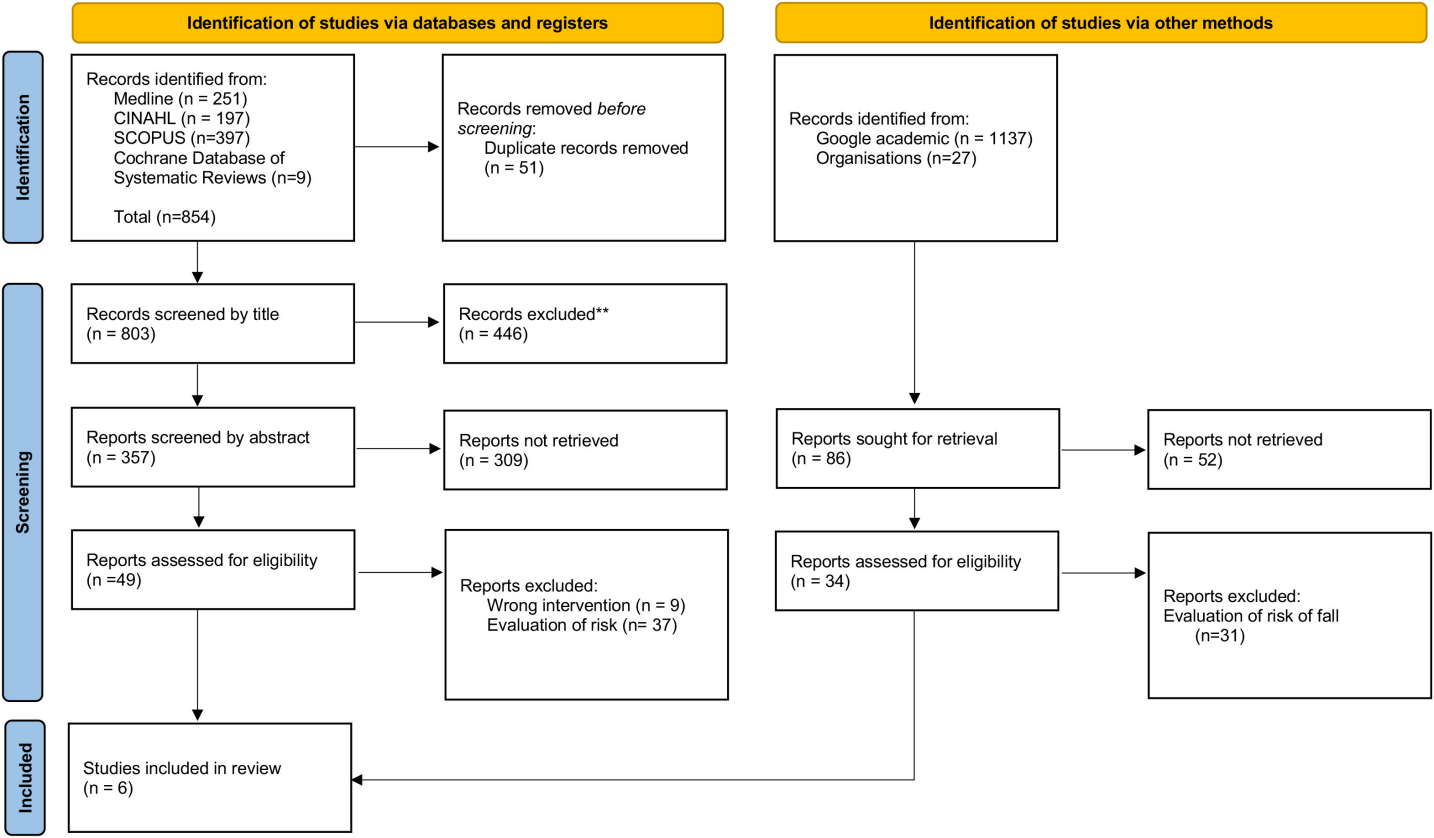
PRISMA-ScR flowchart.

A total of 854 articles were obtained. After removing duplicates (*n* = 51), reading the titles of the articles (*n* = 803), abstracts (*n* = 357), and the complete document (*n* = 49), we identified three articles that allowed answering the investigation question (Figure 1). The articles were thoroughly read. Articles focusing on recording fall risk or prevention interventions were excluded. The search in Google Scholar and the repositories of theses enabled the identification of 34 documents, which after analysis allowed the integration of three guidelines focusing on information to document fall episodes (Figure 1).

Thus, the bibliographic sample of this SR accounts for six documents (Table 2).

**Table 2:** Data extraction.

**Study/Country/Year**	**Study design and aim**	**Results**
Krakau et al. (23) Sweden	Retrospective cohort study The study aimed to validate the nursing documentation, using a specific term in the registered nurses' (RNs) discharge notes regarding inpatient falls according to the outcome of a digitalized data extraction tool and the discharge note itself.	RNs, at discharge, stated that the patient had fallen but no documented evidence of that could be detected during admission. It could also be the opposite, that the RNs correctly had documented that no fall had occurred, but the data extraction tool made an incorrect selection. Information about minor injuries due to the fall was less accurate. In the group where RNs had stated that the patient had fallen without injury, minor injuries had actually occurred in 28.3% of the episodes of care.
Yang et al. (24) Canada	Observational study with video analyses. The study aimed to determine relative risk ratios for hip fractures associated with various fall characteristics.	For each fall, researchers collected data from incident reports completed by nursing staff, which documented the nature and location of injuries from the fall and whether or not the resident was wearing a hip protector. Incident reports also contained data on residents' age, sex, height, weight, and disease diagnoses. The accuracy of incident report data was confirmed through a review of medical records for the 7-day period after the fall.
Montgomery et al. (25) USA	Study of quality improvement The study aimed to implement a system for assessing and documenting patient mobility in an inpatient geriatric unit using a quality improvement framework.	Median daily documentation rates reached 79% by the end of the project. Fall rates did not increase compared to the previous year's baseline (p = 0.80) and the analogous time frames during the previous two years (p = 0.84). Conclusion: A quality improvement framework may be used to improve mobility assessment and documentation in a geriatric unit without increasing patient falls or nursing burden. A review of fall reports revealed that only two out of ten total falls occurred in the presence of nursing staff, and neither were related to mobility assessment or documentation.
Moncada and Mire (26) USA	Create an algorithm for fall risk assessment and interventions	A multifactorial fall risk assessment should be performed for all high-risk persons who require 12 or more seconds to complete the TUG test and report two or more falls or one fall-related injury. The assessment should include circumstances and frequency of falls, associated symptoms, injuries, medications (prescription and over-the-counter), other relevant acute or chronic medical problems, activities of daily living and use of assistive devices, and fear of falling.
National Falls Prevention Coordination Group (27) England	Consensus statement	Older adults reporting a fall or at risk of falling should be observed for balance and gait deficits and considered for risk assessment and risk reduction interventions.
Direção Geral (28) Portugal	Clinical guideline	When a fall happens, immediate evaluation should be carried out by a nurse or physician, and the health unit is responsible for the articulation between the teams and which should include: (a) Assessment of the state of consciousness; (b) Assessment of vital parameters (blood pressure), respiratory rate, heart rate, pain, peripheral oxygen saturation, and capillary glycemia; (c) Assessment of damage associated with the fall; (d) In case of suspected cervical contusion, do not move the person and immobilize the cervical spine; (e) Verification of anticoagulant therapy; (f) Reassessment and monitoring of the clinical situation, taking into account that fall injuries may not arise immediately; (g) Record and description of the fall.

The articles included in this SR show that identifying older adults at risk of falling is the first preventive measure for falls. After a fall, correctly identifying this accident is the first step to preventing its recurrence (23–28).

According to the findings of this review, when a person falls, it is mandatory that the professional who witnessed the fall or to whom the fall was reported, whether by the person himself or by another, registers the notification of the fall while taking into account the following elements (Table 3).

**Table 3:** What to record after a fall?

**Indicator**	**The documentation content**
Who?	Which person fell out? Correct identification of the name and, in case of hospitalization/institutionalization, register unit, number of beds and/or rooms.
What?	What is the clinical situation (state of consciousness, assessment of vital parameters, and determining the intrinsic and extrinsic fall risk)?
When?	Date and time of the fall. In the hospital and nursing homes, record the shift where the fall occurred.
Where?	The exact spot where the fall occurred. It is not enough to mention that it was in the kitchen or the bedroom. We should situate the precise space where the fall occurred and its relationship with the physical elements.
How?	Identify and describe the mechanism of the fall. This description should allow readers to understand how the person fell.
Doing what?	Accurately describe the activity the person was performing when they fell.
What was said?	Describe the person's perceptions regarding the fall, namely, fear of further fall or if it devalues the accident.
What were the consequences?	Record the injuries and their location. Classify any lesion according to the severity, appearance, size, and associated pain. Identify and record changes in the person's behavior after the fall.
What has been done?	Register whether the person needed help to get up/sit/lie down after the fall, if first aid care was provided – which and how, if the nursing care plan was changed, or if there was a change in therapy.

## 4. Discussion

Nurses play a key role in maintaining quality care, for whom the responsibility of documenting in detail and with objectivity makes part (29). Unlike the study by Krakau et al. (30), this review found that the fall episode is not always adequately documented, or lacks detail, due to its own complexity.

Many of the factors assessed by nurses (e.g., data related to symptoms, minor injuries, medication, and limitations in daily living activities) are taken into account before, during, and after the fall event but may not always be recorded. For example, Bjarnadottir and Lucero (31) observed that the documentation made by nurses contains critical information for the clinical evaluation of a fall, but that those factors are not always explicitly mentioned. The nursing records, regarding the event of the fall, present clinical consistency, as identified by this review, including the nature and location of possible injuries, sociodemographic data at the time of the event, vital signs, data about the balance and pattern of walking of the person, as well as an overview of the event itself. Therefore, the documentation of the event can be summarized in five major themes: initial evaluation, nursing diagnoses, care goals, nursing interventions, and evaluation (32).

The documentation concerning the fall event, as well as its prevention and therapeutic intervention, follows an interdependent multifactorial structure. Therefore, there are intrinsic and extrinsic factors that the nurse and the multidisciplinary team should consider in the planning of care. This reality is expressed in some guidelines for preventing falls (33), which can also be used to structure the recording of fall events, namely the fall risk assessment, health literacy, strengthening exercises and balance training, and medical devices. It should be noted that documentation is a fundamental strategy for the prevention of these accidents (8) since many of the people who fall have repeated falls at about the same time while doing the same activities. Therefore, it is necessary to understand the fall pattern so that the subsequent falls can be prevented (8, 9, 19). However, documentation is not always easy to achieve, due to ethical, technological, organizational, social, and individual barriers (34). One solution to better documenting fall events involves making Nursing Information System (NIS) cost-effective and more efficient (35). A recent systematic review (36), with important implications for the management of health services, concluded that the correct use of electronic records to document falls reduces the person's risk and improves the identification of the person and associated factors, increasing the quality of care provided. However, using NIS is not always effective, with some studies indicating the omission of up to one-third of the falls that have occurred (37).

As a result of the rapid implementation of the NIS, there is difficulty in standardizing the data and information recorded, reducing the effectiveness of care, especially prevention. Particularly in the documentation of falls, it can be effective to develop models of information, as suggested by some authors (38), allowing to encode variables and factors related to the person, environment, and organization.

We consider that the results of this SR provide valid indicators to be transposed to the NIS to assist nurses and the multidisciplinary team in the rigorous documentation of fall events.

It should be emphasized that this documentation has increased challenges in the community where the fall events occur in a population that is still active and without physical consequences, and the difficulties of access to healthcare or the devaluation of this type of accident make them difficult to report to health professionals (8, 14, 19, 39). Caregivers and family members should be informed about the importance of reporting falls to health professionals in primary healthcare (40). Underreporting of episodes of falls does not allow for early intervention to prevent recurrence (14).

### 4.1. Strengths and limitations

A strength of this review is that it allows the systematization of what should be recorded after a fall, ensuring that the documentation of these episodes is complete and allowing for the individualization of preventive measures to avoid their recurrence. In contrast, the indicators in Table 3 can be used in computer systems that support the clinical practice of healthcare professionals for the registration and extraction of indicators.

The limitations of this review include language restrictions and free access to full text, and some articles that met the pre-established eligible criteria may have been excluded *a priori*. Another limitation is related to the study's design, which allowed for the mapping of studies on the subject without evaluating their methodological quality.

## 5. Conclusion

The six articles included in the SR allow us to answer the aim of the study. After a fall, registration should include the identification of the person who fell, the time of fall, place, mechanism, activity being performed, the consequences of the fall, and what was done after the fall. Exhaustive documentation of the fall and the fear of falling again is a good clinical practice strategy that allows an understanding of the history of the fall (risk factors, mechanism, and consequences) and the introduction of individualized preventive measures that can prevent its recurrence.

The findings from this review allow us to state that there is no randomized controlled trial that associates the documentation practices depending on the history of falls to the occurrence of second or more adverse events. Therefore, we recommend that future studies evaluate the effectiveness of documentation of episodes of falls in preventing their recurrence, preventive measures adopted, and the control of fear of falls. We also recommend that stakeholders develop training programs that allow healthcare professionals to gain a deeper understanding of documenting falls and use data from these records to create tailored care plans to prevent falls' recurrence.

## Data availability statement

The original contributions presented in the study are included in the article/supplementary material, further inquiries can be directed to the corresponding author.

## Author contributions

CB: conceptualization, resources, supervision, project administration, and funding acquisition. MP, IA, and CB: methodology and software. MP, IA, RB, JB, and CB: validation, data curation, writing—reviewing and editing, investigation, and visualization. MP, IA, RB, and CB: formal analysis and writing—original draft preparation. All authors contributed to the article and approved the submitted version.
